# A deep learning approach for projection and body-side classification in musculoskeletal radiographs

**DOI:** 10.1186/s41747-023-00417-x

**Published:** 2024-02-14

**Authors:** Anna Fink, Hien Tran, Marco Reisert, Alexander Rau, Jörg Bayer, Elmar Kotter, Fabian Bamberg, Maximilian F. Russe

**Affiliations:** 1 Department of Diagnostic and Interventional Radiology, Medical Center – University of Freiburg, Faculty of Medicine, University of Freiburg, Breisacher Str. 64, 79106 Freiburg, Germany; 2 Department of Stereotactic and Functional Neurosurgery, Medical Center – University of Freiburg, Faculty of Medicine, University of Freiburg, Freiburg, Germany; 3 Medical Physics, Department of Diagnostic and Interventional Radiology, Medical Center, University of Freiburg, Faculty of Medicine, University of Freiburg, Freiburg, Germany; 4 Department of Neuroradiology, Medical Center – University of Freiburg, Faculty of Medicine, University of Freiburg, Freiburg, Germany; 5 Department of Trauma and Orthopaedic Surgery, Schwarzwald-Baar Hospital, Villingen-Schwenningen, Germany

**Keywords:** Artificial intelligence, Bone and bones, Deep learning, Musculoskeletal diseases, Radiography

## Abstract

**Background:**

The growing prevalence of musculoskeletal diseases increases radiologic workload, highlighting the need for optimized workflow management and automated metadata classification systems. We developed a large-scale, well-characterized dataset of musculoskeletal radiographs and trained deep learning neural networks to classify radiographic projection and body side.

**Methods:**

In this IRB-approved retrospective single-center study, a dataset of musculoskeletal radiographs from 2011 to 2019 was retrieved and manually labeled for one of 45 possible radiographic projections and the depicted body side. Two classification networks were trained for the respective tasks using the Xception architecture with a custom network top and pretrained weights. Performance was evaluated on a hold-out test sample, and gradient-weighted class activation mapping (Grad-CAM) heatmaps were computed to visualize the influential image regions for network predictions.

**Results:**

A total of 13,098 studies comprising 23,663 radiographs were included with a patient-level dataset split, resulting in 19,183 training, 2,145 validation, and 2,335 test images. Focusing on paired body regions, training for side detection included 16,319 radiographs (13,284 training, 1,443 validation, and 1,592 test images). The models achieved an overall accuracy of 0.975 for projection and 0.976 for body-side classification on the respective hold-out test sample. Errors were primarily observed in projections with seamless anatomical transitions or non-orthograde adjustment techniques.

**Conclusions:**

The deep learning neural networks demonstrated excellent performance in classifying radiographic projection and body side across a wide range of musculoskeletal radiographs. These networks have the potential to serve as presorting algorithms, optimizing radiologic workflow and enhancing patient care.

**Relevance statement:**

The developed networks excel at classifying musculoskeletal radiographs, providing valuable tools for research data extraction, standardized image sorting, and minimizing misclassifications in artificial intelligence systems, ultimately enhancing radiology workflow efficiency and patient care.

**Key points:**

• A large-scale, well-characterized dataset was developed, covering a broad spectrum of musculoskeletal radiographs.

• Deep learning neural networks achieved high accuracy in classifying radiographic projection and body side.

• Grad-CAM heatmaps provided insight into network decisions, contributing to their interpretability and trustworthiness.

• The trained models can help optimize radiologic workflow and manage large amounts of data.

**Graphical Abstract:**

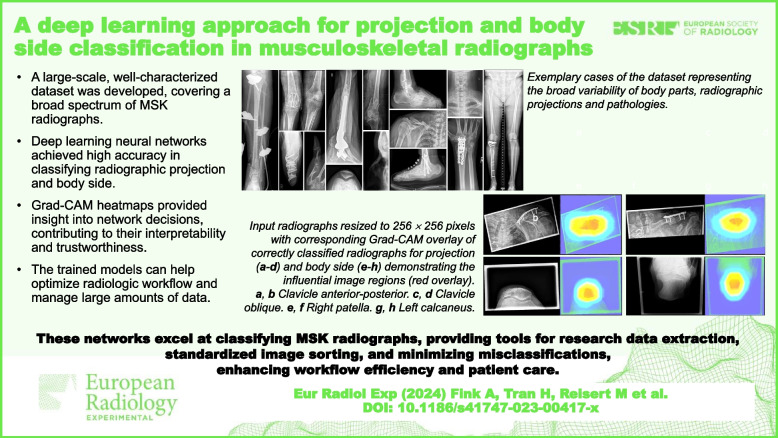

**Supplementary Information:**

The online version contains supplementary material available at 10.1186/s41747-023-00417-x.

## Background

Musculoskeletal diseases impose a high burden on healthcare systems worldwide. The high prevalence of these conditions, combined with the long-term impact of chronic pain and disability after acute treatment, not only diminishes patient well-being but also places a substantial financial load on societies [1]. Customized and appropriate therapy relies on accurate diagnoses and is crucial for the prevention of chronic conditions. Despite the increasing number of cross-sectional computed tomography and magnetic resonance examinations, conventional radiographs still play an indispensable role in the workup of musculoskeletal diseases [2].

Given the rapidly aging population, the prevalence of musculoskeletal conditions is on the rise, leading to a surge in radiological examinations [1, 3]. Consequently, optimizing radiologic workflows becomes paramount, paving the way for supporting artificial intelligence (AI) systems. Numerous models have been developed for the automated identification of pathologies in radiographs, including fracture detection [4, 5], osteoarthritis grading [6], or skeletal maturity assessment [7, 8].

The performance of automated algorithms in pathology detection is significantly enhanced by utilizing larger training datasets [9]. While the Digital Imaging and Communications in Medicine (DICOM) format offers the opportunity to store metadata such as image modality, projection, or side, this information is often inconsistent or missing altogether [10].

To address these constraints and harness image data more effectively, automated metadata classification systems have been proposed. However, existing algorithms primarily focus on classifying body regions [11, 12] or differentiating two singular projections [10, 13].

Operating a multi-classification task, these networks require a substantial amount of training data. While publicly available musculoskeletal datasets exist for singular body regions such as hands [14], knees [15], or upper [16] and lower extremities [17], an open-access dataset encompassing a broad spectrum of all relevant musculoskeletal projections and body regions is currently lacking.

We therefore sought to create a large-scale, well-characterized musculoskeletal radiograph dataset and utilize this training foundation to develop neural networks for the automatic classification of radiographic projection and body side.

## Methods

### Dataset

This retrospective, monocentric study was approved by the local institutional review board (Ethics Committee University of Freiburg: EK:570/19). Informed written consent was waived due to the retrospective study design and patient pseudonymization.

We retrieved all musculoskeletal radiographic studies performed on adult patients between 2018 and 2019 from our institution’s Picture Archiving and Communication System (PACS). To ensure an adequate amount of data for each class, radiographs of rarely examined body regions were also included from the period of 2011 to 2017. These additional body regions comprised the nasal bone, dens, thoracic spine, clavicle, acromioclavicular joint, elbow (radial head), hand, hip, patella, and foot (forefoot, calcaneus, toe). Images of particularly poor quality (not attributable to a radiographic projection, joints destroyed beyond recognition, and incorrectly transferred images) were manually marked and excluded from the dataset.

**Fig. 1:**
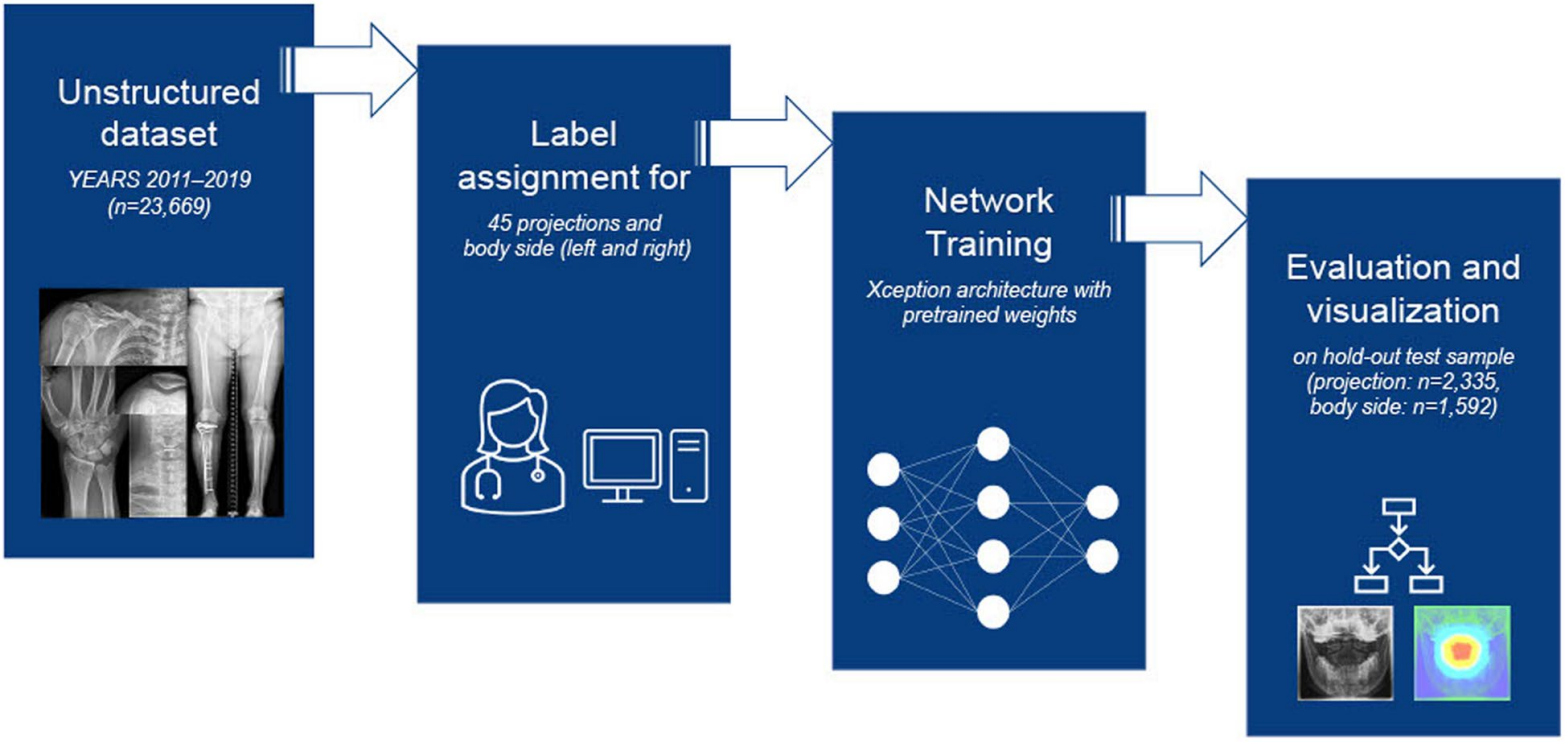
Project workflow from dataset composition, annotation, and network training to final evaluation

**Fig. 2:**
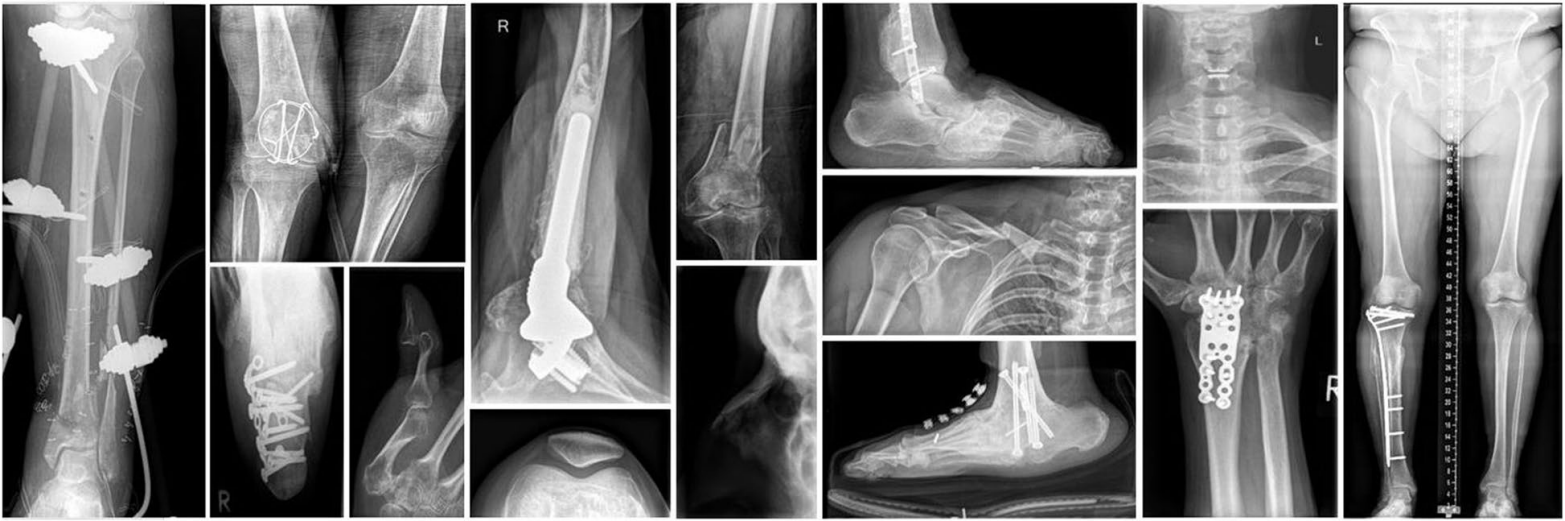
Exemplary cases of the dataset representing the broad variability of body parts, radiographic projections, and pathologies

As a result, a total of 13,098 studies encompassing 23,663 radiographs were included, covering a wide range of musculoskeletal radiology fields with diverse body regions and pathologies as well as radiographs with and without orthopedic implants. The project workflow is depicted in Fig. 1. Figure 2 illustrates a sample selection of the dataset.

To prevent data leakage between training, validation, and test datasets, we only used the first obtained study for each patient within the period of 2011–2019. As some patient studies consisted of multiple individual radiographic projections, a randomized split was performed at the patient level. This resulted in three independent datasets, comprising 19,183 training, 2,145 validation, and 2,335 test images. For side detection, we only included images of paired body regions, leaving a total of 16,319 radiographs and a division into 13,284 training, 1,443 validation, and 1,592 test images.

### Data annotation

Annotation for both network tasks was initially performed by a junior resident (first year of training, A.F.), followed by a consensus reading of uncertain cases with a senior resident (last year of training, H.T.) and a board-certified radiologist (M.F.R.), employing a local instance of the imaging platform Nora [18].

Each x-ray was manually classified according to the represented projection, allocating one of 45 possible machine-readable text labels, a list of which can be found in the supplementary materials (Suppl. 1). Additionally, two labels were assigned to indicate the body side (left or right) on radiographs of paired body regions only. Laterality ground truth was established based on examination notes. This manual classification process, involving initial labeling by a resident followed by a joint evaluation of indeterminate cases with an experienced and a subspecialized trained radiologist, ensured accurate labeling for the subsequent network training.

Table 1 presents an overview of the final dataset for the classification of radiographic projection, displaying the unbalanced label distribution within the dataset with a range from 189 images (toe anterior–posterior [AP]) to 1,267 images (patella tangential). For body-side classification, the dataset was split up into 9,028 images for the left and 7,291 images for the right side, utilizing all available radiographs despite the uneven distribution of examinations for both sides.

**Table 1:** Overview of every depicted projection in the dataset and its frequency of representation

Head/spine		*n*	Arm		*n*	Hand		*n*	Leg		*n*	Foot		*n*
**Nasal bone**	Lateral	268	**AC-joint**	AP	445	**Hand**	AP	487	**Pelvis**	Pelvis AP	833	**Foot**	AP	864
										Hip AP	355		Oblique	771
							Oblique	454						
										Lauenstein	349		Lateral	392
**Cervical spine**	AP	367	**Shoulder**	AP	1,132	**Wrist**	AP	404	**Whole leg**	AP	359	**Forefoot**	AP	306
	Lateral	395		Axial	658									
							Lateral	443					Oblique	560
	Dens	195		Outlet	977									
**Thoracic spine**	AP	659	**Clavicle**	AP	574	**Finger**	AP	333	**Knee**	AP	1,099	**Calcaneus**	Lateral	299
										Lateral	1,157			
	Lateral	620		Oblique	946		Lateral	346						
													Axial	435
										Tangential	1,267			
**Lumbar spine**	AP	604	**Elbow**	AP	358	**Thumb**	AP	208	**Ankle**	AP	720	**Toe**	AP	189
													Lateral	168
				Lateral	384									
	Lateral	608					Lateral	208		Lateral	689	**Big toe**	AP	224
				Radial head	329									
													Lateral	230

*AP* Anterior–posterior, *n* Number of radiographs in the dataset

### Network training

Based on this large-scale labeled dataset, we trained two separate neural networks for the classification of radiographic projection and body side, respectively. Network training was conducted on a standard server graphics processing unit (GPU, Nvidia Tesla RTX A6000). As a deep learning framework, we used the open-source Python library TensorFlow 2.6 [19] and its programming interface Keras [20]. The established network architecture Xception by Chollet et al. [21], originally designed for the classification of multi-colored images with three input channels for the basic colors red, blue, and green, acted as Convolutional Neural Network base. Leveraging this feature, we utilized the original three input channels for each basic color to process our augmented training data.

To optimize the network architecture, adjusting for the reduced number of classes in comparison to the initial network configuration, we removed the top layer and replaced it with a global average pooling layer, a dropout layer to prevent overfitting during training, a dense layer with a rectified linear unit activation function to capture nonlinear dependencies between features and learn complex patterns from the data, and a dense layer with output neurons adapted to the number of classes. The final output decision was determined using a softmax function.

To improve overall network performance and shorten training time, we applied pretrained network weights using the open-access ImageNet database [22]. For training input, we rescaled the variably sized radiographs to a standard network input size of 256 × 256 pixels. To utilize the three input channels of the Xception network, the radiographs were transformed into a three-channel image by incorporating a derived inversion and an edge enhancement image. This approach can improve network performance compared to only using original input radiographs, as shown by Rahman et al. [23]. Edge enhancement was achieved by applying the medianBlur and adaptiveThreshold operations. Training data was augmented using lateral flip and rotation up to 10° for projection training.

To enable body-side detection, the corresponding training process did not involve lateral flip.

We trained both networks for a total of 400 epochs with 300 steps per epoch and a batch size of 15. The initial learning rate started from 0.1 and was gradually reduced to 0.005 using a polynomial decay function.

### Evaluation metrics

We calculated outcome statistics using the Scikit-Learn software library [24]. For statistical analysis, each network output was compared to the manually assigned text label, thus determining model accuracy, precision, and recall. We additionally calculated the Matthews Correlation Coefficient (MCC), which provides a balanced assessment of model accuracy, particularly for unbalanced class distributions. Bootstrapping was used to calculate 95% confidence intervals, which are presented in brackets alongside each metric in the results section.

To address the potential issue of intransparent network predictions, we employed Gradient-weighted Class Activation Mapping (Grad-CAM) [25]. Heatmaps were computed based on the final convolutional layer, providing insight into the specific image regions that influenced the network’s classification decision for every radiograph in the test dataset.

### Code and dataset availability

The model code will be openly accessible as an interactive Jupyter notebook on GitHub. This codebase was created using Python 3.10.12 and leverages framework of TensorFlow 2.13.0, tf-explain 0.3.1, nibabel 4.0.2, cv2 4.8.0, and numpy 1.23.5. It is openly available under the MIT License and can be retrieved from the project’s home page, the XraySorterAI Project (https://github.com/maxrusse/XraySorterAI).

The dataset generated in this study will be provided upon reasonable request, taking into consideration compliance with European data protection regulations and laws.

## Results

### Dataset

The dataset consisted of musculoskeletal radiographs with a mean age of 51.6 years (standard deviation 19.8). The distribution of files by gender was 56% for males and 44% for females.

The x-ray machines used were mainly manufactured by Philips Medical Systems (Hamburg, Germany), to a lesser extent from Samsung Electronics. The datasets are comparable across acquisition technology, x-ray machine manufacturer, spatial resolution, and exposure dosage. A detailed breakdown of the corresponding metadata can be found in the supplementary materials (Suppl. 2–5).

### Radiographic projections

The DICOM-headers used in clinical routine did not contain information on the projection in 28.4% of the 2335 radiographs in the test dataset, emphasizing the necessity of manual labeling for accurate classification within this study. Processing all test images using a single-core server central processing unit (CPU) and no GPU took 139 s, resulting in a classification rate of 16 images/s. The model achieved an overall accuracy of 0.975 (95% confidence interval 0.968–0.981) on the hold-out test sample. Precision measured 0.978 (0.970–0.982), recall 0.973 (0.969–0.981), and MCC 0.974 (0.967–0.981).

Table 2 displays the radiographic projections in which incorrect predictions occurred, along with the corresponding proportion of misclassified radiographs within the overall test dataset. The remaining portion of the test dataset was correctly classified. Among the projections, performance was comparatively lower for the AP view of the clavicle (true positive rate of 0.822) and radial head (true positive rate of 0.800). For a detailed and comprehensive analysis of all network predictions, including true and false positives, the complete confusion matrix can be found in the supplementary materials (Suppl. 6).

**Table 2:** Overview of the radiographic projections in which incorrect network predictions were observed

True label	Prediction	*r*	True label	Prediction	*r*	True label	Prediction	*r*	True label	Prediction	*r*
**Clavicle AP**	Clavicle oblique	0.16	**Hip AP**	Lauenstein	0.04	**Foot AP**	Foot oblique	0.06	**Dens**	Cervical spine AP	0.03
	Shoulder AP	0.02									
**Clavicle oblique**	Clavicle AP	0.07	**Lauenstein**	Hip AP	0.02	**Foot oblique**	Foot AP	0.01	**Lumbar spine AP**	Thoracic spine AP	0.01
	AC-joint	0.01					Forefoot oblique	0.03			
**AC-joint**	Clavicle oblique	0.02	**Knee AP**	Knee lateral	0.01	**Forefoot AP**	Foot AP	0.04	**Lumbar spine lateral**	Lauenstein	0.01
	Shoulder AP	0.02									
**Shoulder AP**	AC-joint	0.04	**Knee lateral**	Elbow AP	0.01	**Forefoot oblique**	Foot oblique	0.09			
	Shoulder outlet	0.03									
**Radial head**	Elbow AP	0.17	**Ankle lateral**	Calcaneus lateral	0.02	**Big toe AP**	Toe AP	0.05			
	Elbow lateral	0.03									
**Hand oblique**	Hand AP	0.05	**Calcaneus lateral**	Ankle lateral	0.07						
**Thumb lateral**	Thumb AP	0.05									

*AP* Anterior–posterior*, r* Relative proportion of misclassified radiographs within the test dataset

Grad-CAM heatmaps provided visual evidence of the image regions that influenced network output decisions. Among the misclassified test images, the most common errors arose from smooth transitions between different projection angles (56%), such as AP and oblique views of the clavicle. Challenges also arose from collimation, mainly making the choice between AP views of the acromioclavicular joint, shoulder, and clavicle (34%) difficult. Metal-dense implant overlay also contributed to classification errors in some cases (5%). In 4% of cases, the exact reason for misclassification remained unclear.

Across all the incorrectly classified test images and 50 randomly selected correctly classified test images, heatmaps consistently highlighted that the image regions influencing network predictions were central parts of the radiograph, such as joint regions or large bone structures.

**Fig. 3:**
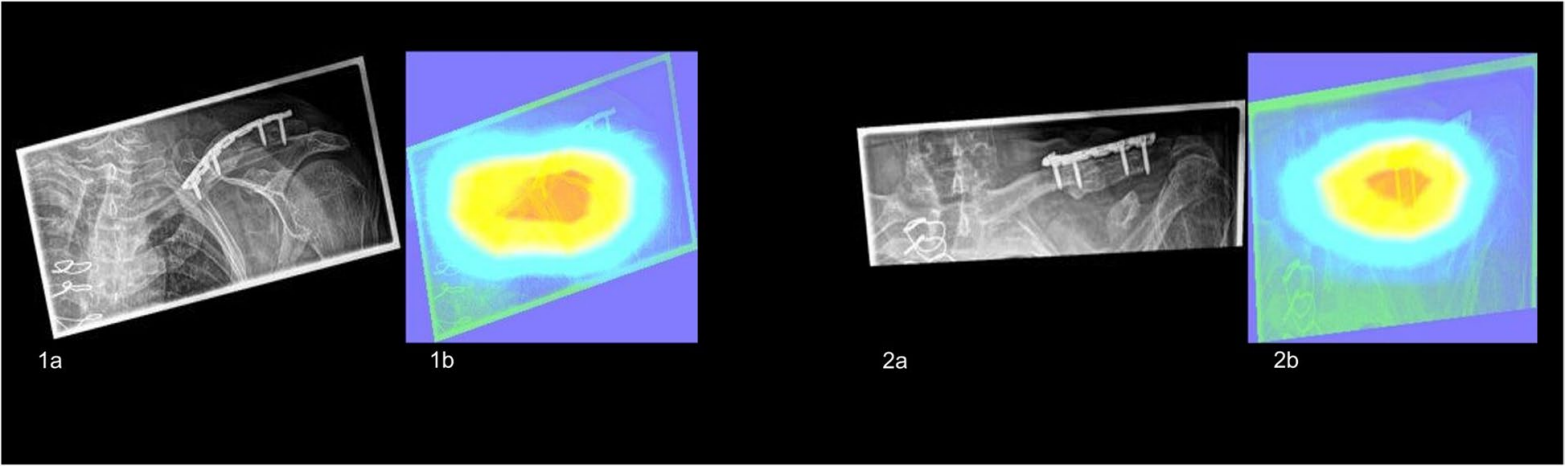
Input radiographs resized to 256 × 256 pixels with corresponding Grad-CAM overlay of two correctly classified projections demonstrating the influential image regions (red overlay). **1a**, **1b** Clavicle anterior–posterior. **2a**, **2b** Clavicle oblique

Figure 3 depicts the heatmaps of two correctly classified radiographs of the clavicle. Exemplary heatmaps illustrating the regions of influence for misclassified projections are provided in the supplementary materials (Suppl. 7).

### Body side

Processing all 1,592 test images using a single-core CPU and no GPU took 48 s, resulting in a classification rate of 33 images per second. The model achieved an overall accuracy of 0.976 (95% confidence interval 0.969–0.983) on the hold-out test sample. Precision measured 0.976 (0.969–0.983), recall 0.976 (0.969–0.983), and MCC 0.973 (0.965–0.981).

Grad-CAM heatmaps were also computed for this task to illustrate which image regions influenced the network’s output decision. Among the misclassified test images, the most common errors were observed in lateral views of single fingers and knees (18% each), followed by AP view of thumb and knee (12% each), lateral view of the foot (9%), and AP view of single fingers and toes (6% each). Closer examination of the misclassifications revealed prominent problems arising from a projection technique inconsistent with our clinic’s SOP, such as inverted radiation beam path or body part position (48%), alongside challenges posed by metal-dense implants (15%) and unusual pathologies such as foot amputation (6%). In 24% of cases, the exact reason for misclassification remained unclear, mainly involving lateral views of individual fingers.

Across all the incorrectly classified test images and 50 randomly selected correctly classified test images, the heatmaps consistently highlighted that the network’s output decision was centered on crucial image areas, particularly joint gaps. Notably, none of the heatmaps focused on the sometimes visually displayed side labels “L” and “R,” as visualized in the sample heatmaps provided in the supplementary materials (Suppl. 8).

**Fig. 4:**
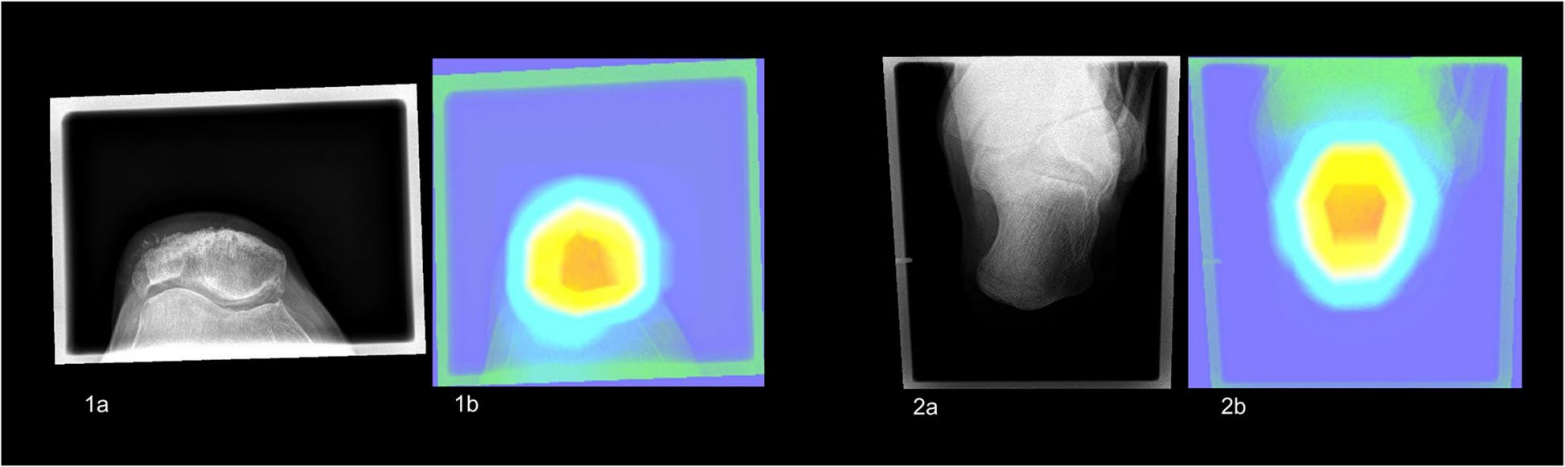
Input radiographs resized to 256 × 256 pixels with corresponding Grad-CAM overlay of two correctly classified radiographs for the body side demonstrating the influential image regions (red overlay). **1a**, **1b** Right patella. **2a**, **2b** Left calcaneus

Figure 4 provides two examples of heatmaps representing correctly classified radiographs, highlighting the influential regions. Supplementary materials contain additional heatmaps showcasing instances of incorrect classifications (Suppl. 9).

## Discussion

We developed a large-scale, well-characterized dataset of musculoskeletal radiographs and trained corresponding networks for the classification of radiographic projection and body side. The models exhibited excellent and fast performance, achieving an accuracy of 0.975 for projection and 0.976 for body-side classification. The models’ robustness was further highlighted by their performance on an unknown test dataset containing radiographs with various underlying pathologies and orthopedic implants. Moreover, the utilization of Grad-CAM heatmaps provided an additional layer of interpretability by visualizing the image regions that influenced the model’s output decisions.

In the context of rapidly increasing examination numbers, it is crucial to organize and validate both radiographs and their associated metadata, particularly considering the prevalent inconsistencies or lack of image-related metadata in DICOM-headers. Previous studies have emphasized the importance of large labeled datasets for neural network training, such as the MURA dataset for the upper extremity (40,561 images [16]) and the LERA dataset for the lower extremity (93,455 images [17]) provided by the Stanford Machine Learning Group. The release of both datasets each prompted multiple subsequent projects focusing on abnormality detection in musculoskeletal radiographs [26–28]. However, these datasets primarily focused on presorting body regions, assigning labels at study and patient levels, respectively. Our dataset stands out for its comprehensive coverage of musculoskeletal radiographs, encompassing a broader spectrum of images than previously available datasets. This breadth allows our models to handle multiclassification tasks across a wide range of body regions, with 45 distinct labels for radiographic projection and additional differentiation of body side. The dataset’s high quality was further ensured by involving three distinct labelers, including a resident and two experienced radiologists, in the manual classification process.

Previous studies on sorting networks primarily focused on classifying musculoskeletal radiographs based on broader body regions [11, 12]. In contrast, our approach takes a step further by classifying radiographs based on their precise projection and body side. Compared to related studies that primarily focused on distinguishing two chest x-ray projections [10, 13] or classifying radiographs into 30 categories [29], our models demonstrate the ability to classify radiographs across a wide range of 45 different projections while also incorporating body side detection, outperforming the previous research in terms of accuracy and scope, respectively.

In our study, projections with unique features, such as nasal bone or whole leg AP, achieved excellent classification rates. Errors were infrequent and occurred primarily in projections such as the AP view of the clavicle (often misclassified as clavicle oblique) and the radial head (often misclassified as AP elbow). In clinical practice, these projections are often affected by non-orthograde adjustment techniques and show a seamless anatomical transition to other views. Similarly, body-side detection errors were more prevalent in radiographs of single fingers and toes or the tangential view of the patella, where distinguishing the body side is subjectively challenging. Nonetheless, our models demonstrated success in accurately distinguishing even these challenging classes, resulting in overall accuracies comparable to previous studies [13].

The incorporation of Grad-CAM heatmaps in our analysis enhanced the interpretability and transparency of the network’s outputs, addressing the inherent “black box” nature of neural networks with multiple hidden layers. By visualizing the image regions that played a decisive role in the output, we showed that the network’s decisions aligned with human viewers’ interpretations. Even for the majority of incorrect predictions, we managed to make network decisions understandable. The influential regions identified by the heatmaps often corresponded to clinically relevant areas such as the joint space or prominent bone structures.

Furthermore, our findings demonstrated that the network’s body-side classification was not reliant on the visually depicted side labels “L” and “R”, as a human viewer would interpret. Instead, the classification was primarily based on bone structures within the radiographs. It is noteworthy that the side label was not always a physical opaque marker added by the technologist prior to imaging but rather often a digital overlay within the PACS, and thus not directly encoded in the raw data accessible to the network.

Despite these promising results, our study has limitations. Given the large number of classes in the projection training, class balancing was not feasible. Nevertheless, the substantial number of radiographs per class allowed for an excellent classification accuracy. This finding is consistent with previous studies, where increasing data volume significantly improved precision and recall, while balancing techniques barely showed any improvement [9].

As the study was monocentric and retrospective in nature, we did not have the opportunity to validate the trained models on radiographs from external institutions. To mitigate this, we implemented a randomized dataset split on a patient level, creating a hold-out test sample that was unknown to the models. Furthermore, we took measures to create a highly heterogeneous dataset that encompasses radiographs from everyday clinical practice. This dataset was obtained from various examiners, captured using different devices, and depicted a wide range of pathologies and orthopedic implants. We believe that the excellent performance of our models on such a diverse dataset suggests their applicability to external datasets, but further validation through external studies is warranted.

In summary, the developed networks exhibited exceptional performance in classifying a wide range of musculoskeletal radiographs, enabling precise data extraction in research and automated image sorting for standardized reporting. Implementing them as pre-sorting algorithms for end-to-end solutions targeted on specific body regions showcases the great potential for minimizing misclassifications, ultimately enhancing radiology workflow efficiency and patient care.

### Supplementary Information

**Additional file 1: Suppl. 1.  **List of all labels used in annotating training data for projection classification (*AP*: anterior-posterior). **Suppl. 2. **Image metadata on acquisition technology, x-ray machine manufacturer, and spatial resolution, for the training dataset (*CR*: computed radiography, *DX*: digital x-ray). **Suppl. 3. **Image metadata on acquisition technology, x-ray machine manufacturer, and spatial resolution for the validation dataset (*CR*: computed radiography, *DX*: digital x-ray). **Suppl. 4. **Image metadata on acquisition technology, x-ray machine manufacturer, and spatial resolution for the test dataset (*CR*: computed radiography, *DX*: digital x-ray). **Suppl. 5. **Image metadata on exposure dose in kVp and mAS for the training, validation, and test dataset (*kVp*: kilovoltage peak, *mAS*: milliampere-seconds).** Suppl. 6. **Normalized confusion matrix for the classification of 45 distinct radiographic projections. **Suppl. 7. **Input radiographs resized to 256 x 256 pixels with corresponding Grad-CAM overlay of wrongly classified projections, demonstrating the influential image regions (red overlay). 1a/b: clavicle AP (prediction: shoulder AP), 2a/b: dens (prediction: c-spine AP), 3a/b: knee lateral (prediction: elbow lateral), 4a/b: l-spine AP (prediction: t-spine AP). **Suppl. 8.  **Input radiographs resized to 256 x 256 pixels with corresponding Grad-CAM overlay of correctly classified radiographs for body side with a visually displayed radiopaque side marker (1a/b: right hand. 2a/b: right thumb. 3a/b: right shoulder AP, 4a/b: right toe), demonstrating the influential image regions (red overlay). **Suppl. 9. **Input radiographs resized to 256 x 256 pixels with corresponding Grad-CAM overlay of left body parts wrongly classified as right body side (1a/b: foot, 2a/b: knee, 3a/b: thumb, 4a/b: knee), demonstrating the influential image regions (red overlay).

## Data Availability

The model code will be openly accessible as an interactive Jupyter notebook on GitHub. This codebase was created using Python 3.10.12 and leverages framework of TensorFlow 2.13.0, tf-explain 0.3.1, nibabel 4.0.2, cv2 4.8.0, and numpy 1.23.5. It is openly available under the MIT License and can be retrieved from the project’s home page, the XraySorterAI Project (https://github.com/maxrusse/XraySorterAI).

## Abbreviations

AI: Artificial intelligence
AP: Anterior–posterior
CPU: Central processing unit
DICOM: Digital Imaging and Communications in Medicine
GPU: Graphics processing unit
Grad-CAM: Gradient-weighted class activation mapping
MCC: Matthews correlation coefficient
PACS: Picture Archiving and Communication System
