# Supplementary material for: A deep learning approach for projection and body-side classification in musculoskeletal radiographs
Source: Eur Radiol Exp. 2024 Feb 14;8:23. doi: 10.1186/s41747-023-00417-x (PMC10866807; doi:10.1186/s41747-023-00417-x)
Supplement: Supplementary file 1 — Additional file 1: Suppl. 1. List of all labels used in annotating training data for projection classification (AP: anterior-posterior). Suppl. 2. Image metadata on acquisition technology, x-ray machine manufacturer, and spatial resolution, for the training dataset (CR: computed radiography, DX: digital x-ray). Suppl. 3. Image metadata on acquisition technology, x-ray machine manufacturer, and spatial resolution for the validation dataset (CR: computed radiography, DX: digital x-ray). Suppl. 4. Image metadata on acquisition technology, x-ray machine manufacturer, and spatial resolution for the test dataset (CR: computed radiography, DX: digital x-ray). Suppl. 5. Image metadata on exposure dose in kVp and mAS for the training, validation, and test dataset (kVp: kilovoltage peak, mAS: milliampere-seconds). Suppl. 6. Normalized confusion matrix for the classification of 45 distinct radiographic projections. Suppl. 7. Input radiographs resized to 256 x 256 pixels with corresponding Grad-CAM overlay of wrongly classified projections, demonstrating the influential image regions (red overlay). 1a/b: clavicle AP (prediction: shoulder AP), 2a/b: dens (prediction: c-spine AP), 3a/b: knee lateral (prediction: elbow lateral), 4a/b: l-spine AP (prediction: t-spine AP). Suppl. 8. Input radiographs resized to 256 x 256 pixels with corresponding Grad-CAM overlay of correctly classified radiographs for body side with a visually displayed radiopaque side marker (1a/b: right hand. 2a/b: right thumb. 3a/b: right shoulder AP, 4a/b: right toe), demonstrating the influential image regions (red overlay). Suppl. 9. Input radiographs resized to 256 x 256 pixels with corresponding Grad-CAM overlay of left body parts wrongly classified as right body side (1a/b: foot, 2a/b: knee, 3a/b: thumb, 4a/b: knee), demonstrating the influential image regions (red overlay). [file 41747_2023_417_MOESM1_ESM.docx]

## **A deep learning approach for projection and body side classification in musculoskeletal radiographs**

## **ELECTRONIC SUPPLEMENTARY MATERIAL**

| **Radiographic projection** | **Label** |
| --- | --- |
| Nasal Bone lateral | nasal_bone |
| Cervical Spine AP Cervical Spine lateral Dens Thoracic Spine AP Thoracic Spine lateral Lumbar Spine AP Lumbar Spine lateral | ap_cspine lat_cspine dens ap_tspine lat_tspine ap_lspine lat_lspine |
| AC-joint Shoulder AP Shoulder outlet Shoulder axial Clavicle AP Clavicle oblique | ap_ac ap_shoulder y-view axial_shoulder ap_clavicle obl_clavicle |
| Elbow AP  Elbow lateral Radial Head | ap_elbow lat_elbow radial_head |
| Hand AP Hand oblique Wrist AP Wrist lateral Finger AP Finger lateral Thumb AP Thumb lateral | ap_hand obl_hand ap_wrist lat_wrist ap_finger lat_finger ap_thumb lat_thumb |
| Pelvis AP Hip AP Lauenstein | ap_pelvis ap_hip Lauenstein |
| Whole Leg AP | ap_leg |
| Knee AP Knee lateral Patella tangential | ap_knee lat_knee defile |
| Ankle AP Ankle lateral Calcaneus lateral Calcaneus axial Foot AP Foot lateral Foot oblique Forefoot AP Forefoot oblique Toe AP Toe lateral Big Toe AP Big Toe lateral | ap_ankle lat_ankle lat_calcaneus axial_calcaneus ap_foot lat_foot obl_foot ap_forefoot obl_forefoot ap_toe lat_toe ap_btoe lat_btoe |

**Suppl. 1:** List of all labels used in annotating training data for projection classification (*AP*: anterior-posterior).

|  | **Acquisition Technology** | **Manufacturer** | **Spatial Resolution** |
| --- | --- | --- | --- |
| DX | 0,9989 |  |  |
| CR | 0,0011 |  |  |
| Philips Medical Systems |  | 0,8773 |  |
| Samsung Electronics |  | 0,1227 |  |
| 0,144 |  |  | 0,3579 |
| 0,143 |  |  | 0,3151 |
| 0,148 |  |  | 0,1495 |
| 0,14 |  |  | 0,1229 |
| 0,100000001 |  |  | 0,0351 |
| 0,15 |  |  | 0,0167 |
| 0,2 |  |  | 0,0027 |

**Suppl. 2:** Image metadata on acquisition technology, x-ray machine manufacturer, and spatial resolution, for the training dataset (*CR*: computed radiography, *DX*: digital x-ray).

|  | **Acquisition Technology** | **Manufacturer** | **Spatial Resolution** |
| --- | --- | --- | --- |
| DX | 0,9977 |  |  |
| CR | 0,0023 |  |  |
| Philips Medical Systems |  | 0,8771 |  |
| Samsung Electronics |  | 0,1229 |  |
| 0,144 |  |  | 0,3631 |
| 0,143 |  |  | 0,3092 |
| 0,148 |  |  | 0,1512 |
| 0,14 |  |  | 0,1235 |
| 0,100000001 |  |  | 0,0373 |
| 0,15 |  |  | 0,0101 |
| 0,2 |  |  | 0,0055 |

**Suppl. 3** Image metadata on acquisition technology, x-ray machine manufacturer, and spatial resolution for the validation dataset (*CR*: computed radiography, *DX*: digital x-ray).

|  | **Acquisition Technology** | **Manufacturer** | **Spatial Resolution** |
| --- | --- | --- | --- |
| DX | 0,9979 |  |  |
| CR | 0,0021 |  |  |
| Philips Medical Systems |  | 0,8613 |  |
| Samsung Electronics |  | 0,1387 |  |
| 0,144 |  |  | 0,3278 |
| 0,143 |  |  | 0,3181 |
| 0,148 |  |  | 0,1448 |
| 0,14 |  |  | 0,1389 |
| 0,100000001 |  |  | 0,0444 |
| 0,15 |  |  | 0,0222 |
| 0,2 |  |  | 0,0038 |

**Suppl. 4:** Image metadata on acquisition technology, x-ray machine manufacturer, and spatial resolution for the test dataset (*CR*: computed radiography, *DX*: digital x-ray).

| **Dataset** | **Value** | **kVp** | **mAS** |
| --- | --- | --- | --- |
| Training | Median | 66.0 | 4,100.0 |
|  | 95% Confidence Interval (Median) | [66.0; 66.0] | [3,900.0; 4,300.0] |
| Validation | Median | 65.9 | 3,900.0 |
|  | 95% Confidence Interval (Median) | [63.0; 66.0] | [3,200.0; 4,100.0] |
| Test | Median | 66.0 | 4,315.0 |
|  | 95% Confidence Interval (Median) | [66.0; 66.0] | [3,900.0; 4,500.0] |

**Suppl. 5:** Image metadata on exposure dose in kVp and mAS for the training, validation, and test dataset (*kVp*: kilovoltage peak, *mAS*: milliampere-seconds).


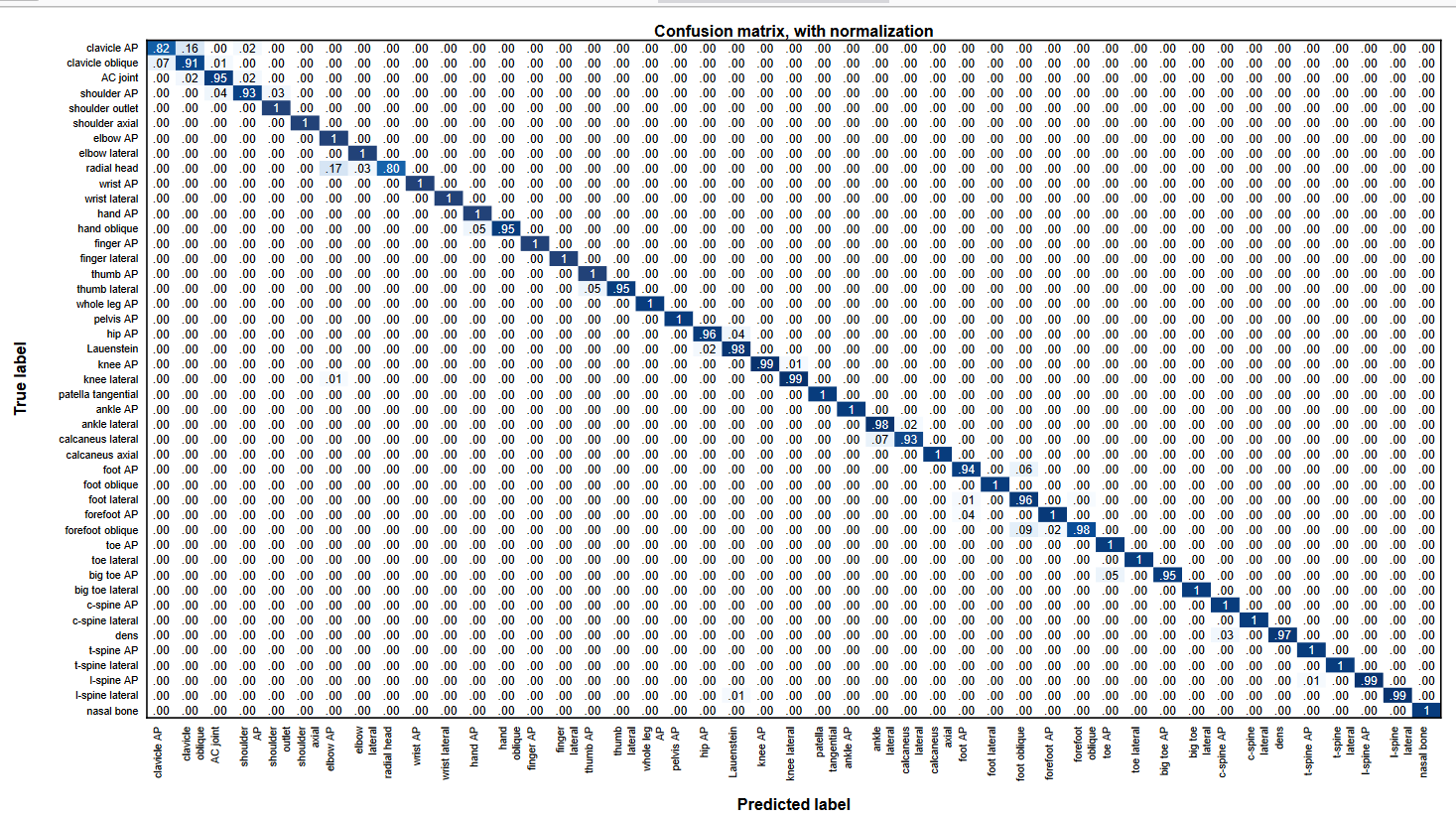


**Suppl. 6:** Normalized confusion matrix for the classification of 45 distinct radiographic projections.


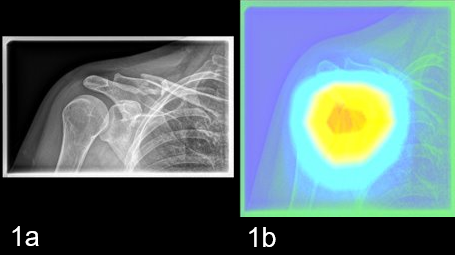

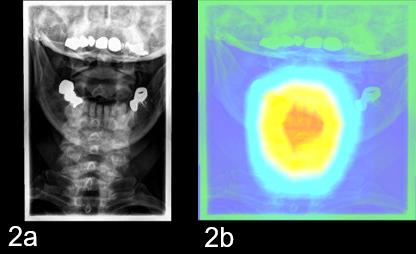


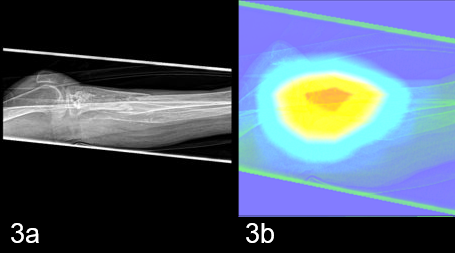

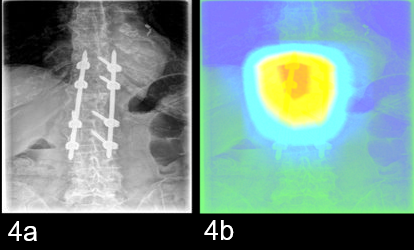


**Suppl. 7:** Input radiographs resized to 256 x 256 pixels with corresponding Grad-CAM overlay of wrongly classified projections, demonstrating the influential image regions (red overlay). 1a/b: clavicle AP (prediction: shoulder AP), 2a/b: dens (prediction: c-spine AP), 3a/b: knee lateral (prediction: elbow lateral), 4a/b: l-spine AP (prediction: t-spine AP).

*
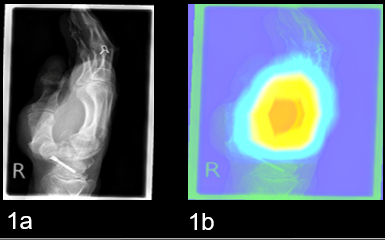
* *
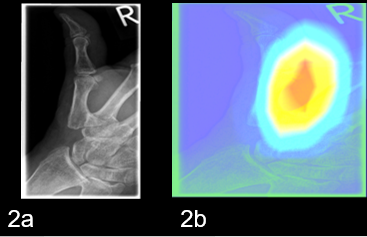
*
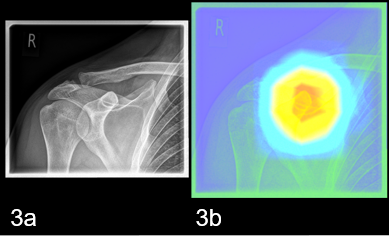

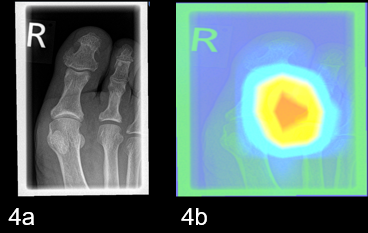


**Suppl. 8:** Input radiographs resized to 256 x 256 pixels with corresponding Grad-CAM overlay of correctly classified radiographs for body side with a visually displayed radiopaque side marker (1a/b: right hand. 2a/b: right thumb. 3a/b: right shoulder AP, 4a/b: right toe), demonstrating the influential image regions (red overlay).


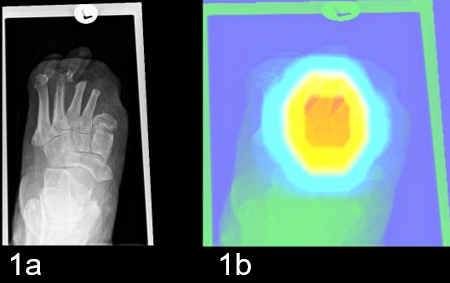

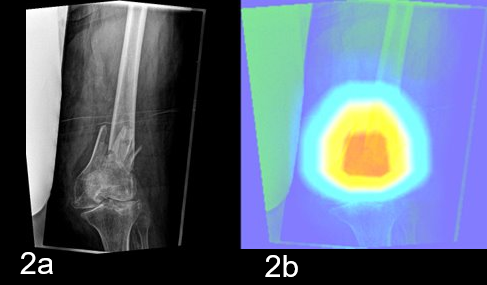


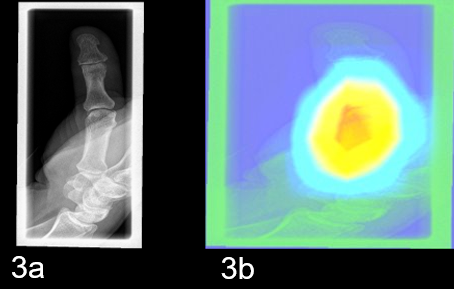

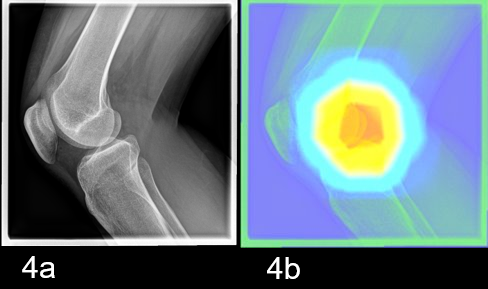


**Suppl. 9:** Input radiographs resized to 256 x 256 pixels with corresponding Grad-CAM overlay of left body parts wrongly classified as right body side (1a/b: foot, 2a/b: knee, 3a/b: thumb, 4a/b: knee), demonstrating the influential image regions (red overlay).
